# Whether a polyethylene terephthalate bottle cap can be opened could serve as a common indicator for locomotive syndrome, frailty and sarcopenia

**DOI:** 10.1111/ggi.70076

**Published:** 2025-05-21

**Authors:** Yohei Sawaya, Tamaki Hirose, Masahiro Ishizaka, Naori Hashimoto, Akira Kubo, Tomohiko Urano

**Affiliations:** ^1^ Department of Physical Therapy, School of Health Sciences International University of Health and Welfare Otawara Japan; ^2^ Senior Services Division of Otawara Otawara Japan; ^3^ Department of Physical Therapy School of Health Sciences at Odawara, International University of Health and Welfare Odawara Japan; ^4^ Department of Geriatric Medicine School of Medicine, International University of Health and Welfare Chiba Japan

**Keywords:** frailty, Japan, polyethylene terephthalates, sarcopenia, screening

## Abstract

**Aim:**

This study aimed to develop an easy‐to‐use assessment that can be carried out anywhere by investigating the association between the ability to open a polyethylene terephthalate (PET) bottle cap and locomotive syndrome (LS), frailty and sarcopenia, and the screening ability of this method.

**Methods:**

This cross‐sectional study was carried out between July 2022 and February 2024, analyzing 341 community‐dwelling Japanese older adults (mean age 80.0 ± 6.9 years). We used odds ratios (OR) and their 95% confidence intervals (CI) to assess the association between the ability to open a PET bottle cap and LS, frailty and sarcopenia, as well as the screening ability of this method. LS was evaluated using the stand‐up and two‐step tests and Geriatric Locomotive Function Scale‐5; frailty was evaluated using the Japanese version of the Cardiovascular Health Study criteria; and sarcopenia was evaluated using grip strength, walking speed and skeletal muscle mass index.

**Results:**

After adjusting for age, sex and body mass index, the binary logistic regression analysis showed that the inability to open a PET bottle cap was associated with LS stages 2 and 3 (OR 2.72, 95% CI 1.46–5.07), frailty according to the Japanese version of the Cardiovascular Health Study criteria (OR 3.38, 95% CI 1.23–9.24) and sarcopenia (OR 3.61, 95% CI 1.67–7.79). The receiver operating characteristic curve analysis showed moderate screening ability for LS, frailty and sarcopenia, with a positive predictive value of 97.4% for LS, and 93.9% for pre‐frailty and frailty.

**Conclusions:**

Notably, the positive predictive value was high for LS, and pre‐frailty/frailty using the PET bottle method. The PET bottle method is “simple” and requires just “one action,” and it holds promise for practical social implementation as a comprehensive assessment of LS (stages 2 and 3), frailty and sarcopenia. **Geriatr Gerontol Int 2025; 25: 905–910**.

## Graphical abstract

This study demonstrated that the inability to open a polyethylene terephthalate (PET) bottle cap was associated with a 2.72‐fold increased risk of locomotive syndrome (LS) stages 2 and 3, 3.38‐fold for frailty, and 3.61‐fold for sarcopenia. Moreover, this inability showed potential as a simple case‐finding tool for identifying individuals at risk of LS (stages 2 and 3), frailty, and sarcopenia.

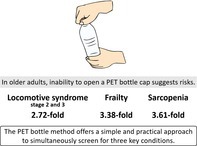

## Introduction

In 2022, the Japanese Medical Science Federation issued the “Declaration of the Medical Society for Overcoming Frailty and Locomotive Syndrome” to promote healthy longevity in the Japanese population.^1^, ^2^ Frailty is characterized by weakened resistance and decreased physical strength as a result of aging. It comprehensively addresses physical, mental, psychological and social aspects, thus providing a multidimensional understanding of older adults.^3^ Locomotive syndrome (LS) is a condition characterized by impaired mobility functions, including standing and gait, primarily caused by disorders of the musculoskeletal system.^4^, ^5^ Additionally, sarcopenia, which refers to an age‐related decrease in muscle mass and strength, is considered the core of physical frailty, with a primary focus on muscles.^6^, ^7^ The perspectives of these three concepts are different, and each has its own independent assessment criteria; however, they are deeply interconnected with each other.^8^, ^9^ Additionally, these three concepts are reversible and share the important common goal of recovery.^1^, ^2^, ^10^, ^11^ Therefore, prevention at an early stage of life is crucial. Therefore, to achieve early detection and intervention for LS, frailty, and sarcopenia, which share many common features, it is essential to develop an integrated assessment method that recognizes early symptoms. An integrated assessment approach can efficiently identify high‐risk individuals while minimizing resource utilization, thereby facilitating appropriate interventions. Additionally, this method should be accessible not only to healthcare professionals, but also to older adults and their families, allowing them to easily carry it out in daily life, anywhere and anytime.

Therefore, we used polyethylene terephthalate (PET) bottles. In Japan, most people of all ages and sexes consume beverages from PET bottles, which are ubiquitous and easily accessible in their daily lives. Furthermore, the recycling rate of PET bottles in Japan reached 86.0% in 2022, which was exceptionally high compared with other countries.^12^ Considering these circumstances, we developed a PET bottle method to evaluate the ability required to open a cap through actual actions and questionnaires. The cutoff values for grip strength, determined by the ability required to open the PET bottle cap, were 17.7 kg based on actual actions and 20.5 kg based on questionnaires.^13^, ^14^ Additionally, we clarified that the difference in whether the PET bottle cap can be opened immediately, as determined by the self‐reported questionnaire, reflected the multifaceted nature of frailty.^15^

Based on these findings, we hypothesized that the ability to open a PET bottle cap is related not only to sarcopenia and frailty, but also to LS, which has not been clarified in previous studies. Furthermore, as far as we are aware, no study has attempted to simultaneously and comprehensively assess LS, frailty and sarcopenia using activities of daily living. A comprehensive evaluation approach based on simple and familiar tasks, such as the PET bottle method, might effectively enhance individuals' awareness of their own risks and encourage behavioral modifications. Accordingly, the present study aimed to investigate the association and screening ability between the actual ability to open a PET bottle cap and LS, frailty and sarcopenia, and to clarify the usefulness of PET bottle methods in detecting physical and mental decline.

## Methods

### Study design

This cross‐sectional study was carried out between July 2022 and February 2024. Each participant was provided with both oral explanations and written details of the study protocol, and written informed consent was obtained from all participants. The Ethics Committee of the International University of Health and Welfare (Approval No. 18‐Io‐158‐2) approved this study, which adhered to the principles outlined in the Declaration of Helsinki.

### Study setting and participants

**Figure 1:**
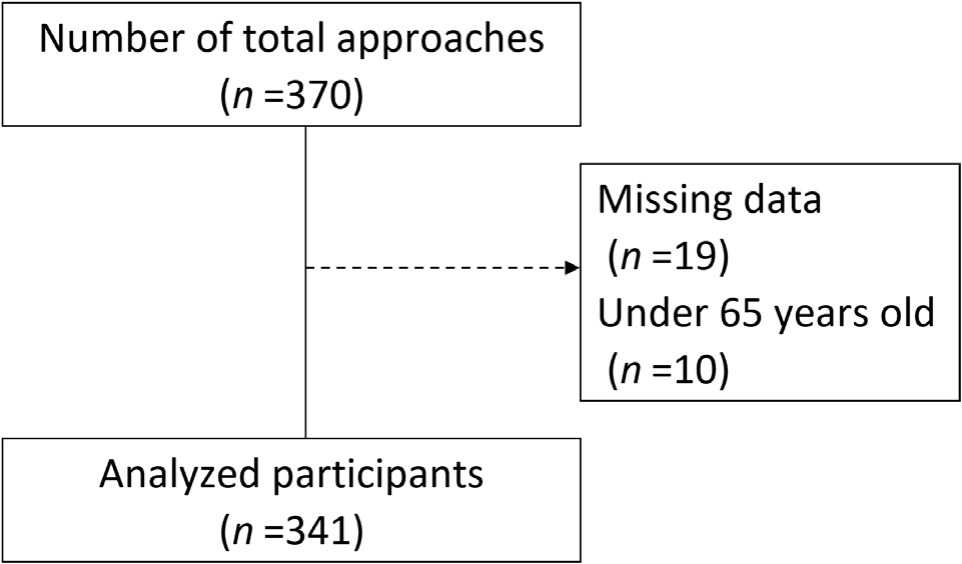
Flow chart of research participants.

The participants were 370 residents of City A in the Tochigi Prefecture. Recruitment was carried out by the city hall contacting representatives of “Kayoi‐no‐ba,” who then reached out to potential participants.^16^ Alternatively, information could be provided in the city's public relations magazine. The participants voluntarily participated in a city‐sponsored caregiving prevention program. After excluding individuals with missing data and those aged <65 years, 341 participants (average age 80.0 ± 6.9 years, 60 men and 281 women) were included in the analysis (Fig. 1). The dataset of this study does not overlap with research on PET bottle cap opening that focuses on sarcopenia and frailty as the main outcomes.^13^, ^15^

### PET bottle cap opening test

Participants carried out the task of opening the cap of an unopened 525‐mL PET bottle (Oi Ocha; ITO EN, Tokyo, Japan) while seated. The following instructions were given for opening the bottle: “Open it as you usually would,” “Hold the PET bottle with one hand and the cap with the other,” “Do not hold the bottle with your thighs” and “Open it without spilling the tea.” The participants were allowed to choose the hand to be used to open the cap. They were given approximately 30 s to complete the task. Staff from the caregiving prevention program (e.g. nurses, public health nurses and physical therapists) determined whether the cap was successfully opened. Participants were classified into Success and Failure groups based on their ability to open the bottle.

### Locomotive syndrome

LS was assessed using three tests: the stand‐up test, the two‐step test and the 5‐question Geriatric Locomotive Function Scale (GLFS‐5). Each test was carried out as in previous studies.^2^, ^17^, ^18^ If an individual could not stand up from a 40‐cm high platform using either leg in the stand‐up test, had a two‐step test value of <1.3 or scored ≥6 on the GLFS‐5, they were judged to have LS.^2^, ^17^, ^18^ LS stages 1, 2 and 3 were determined based on the more severe stages of the stand‐up and two‐step test.^2^, ^17^

### Frailty

#### Primary assessment

Frailty was assessed using the revised Japanese version of the Cardiovascular Health Study criteria (J‐CHS).^19^ The criteria for assessment were as follows: J‐CHS scores of 0 were classified as robust, 1–2 as pre‐frailty and ≥3 as frailty.^19^

#### Secondary assessments

Frailty was assessed using the Questionnaire for Medical Checkup of Old‐Old (QMCOO) and Kihon Checklist (KCL).^20^, ^21^ The criteria for assessment were as follows: QMCOO scores of 0–2 were classified as robust, 3 as pre‐frailty and ≥4 as frailty; and KCL scores of 0–3 were classified as robust, 4–7 as pre‐frailty and ≥8 as frailty.^21^, ^22^, ^23^, ^24^ QMCOO and KCL are comprehensive geriatric assessments, whereas J‐CHS evaluates the phenotype model.^3^, ^19^

### Sarcopenia

Sarcopenia was determined based on grip strength, usual walking speed and skeletal muscle mass index. Grip strength was assessed using a Smedley‐type dynamometer (TKK 5401 Grip‐D; Takei Scientific Instruments, Niigata City, Japan), with the maximum value obtained from one measurement on each side while standing considered the representative value. The usual walking speed was measured once on a 4‐ or 5‐m walkway equipped with acceleration and deceleration zones. Skeletal muscle mass index was evaluated using the bioelectrical impedance analysis method with a body composition analyzer (MC‐780A/A‐N; TANITA, Tokyo, Japan). The diagnostic criteria and cut‐off values for sarcopenia were based on the Asian Working Group on Sarcopenia 2019 guidelines.^6^ Sarcopenia was defined as low skeletal muscle mass combined with low muscle strength and/or low physical function, and severe sarcopenia was defined as all three measurement values being below the reference values.^6^

### Statistical analysis

Participants were divided into the Success group and the Failure group, and group comparisons were carried out using independent *t*‐tests, Mann–Whitney *U*‐tests and χ^2^‐test/Fisher's exact test as appropriate. In the binary logistic regression analysis, LS, frailty and sarcopenia were set as dependent variables; the ability to open a PET bottle cap was set as the independent variable; and age, sex and body mass index were set as adjustment variables. Due to the potential impact of age, sex and body mass index on the prevalence of LS, frailty and sarcopenia, and their role as confounding variables, these factors were included as covariates in the analysis. This approach is generally consistent with previous studies that also used these factors as covariates when analyzing factors associated with LS, frailty and sarcopenia.^24^, ^25^, ^26^, ^27^, ^28^ This analysis calculated the odds ratios (OR) and their 95% confidence intervals (CI) for LS, frailty and sarcopenia associated with the inability to open a bottle cap. We evaluated the sensitivity, specificity, positive predictive value (PPV), negative predictive value and area under the curve (AUC) of the screening tool based on the ability required to open a PET bottle cap to detect LS, frailty and sarcopenia using receiver operating characteristic (ROC) analysis. In the sex‐specific analyses, men were analyzed using group comparisons, whereas women were analyzed using group comparisons, binary logistic regression and ROC analysis. Statistical analyses were carried out using IBM SPSS version 25 (IBM Japan, Tokyo, Japan), with a significance level of 5%.

## Results

The Success group included 265 participants (77.7%), whereas the Failure group included 76 participants (22.3%). Group comparisons between the Success and Failure groups for LS, the J‐CHS criteria and sarcopenia are presented in Table 1. Similar group comparisons for the QMCOO and KCL are shown in Table S1. Comparisons between groups showed significant differences in all assessment indicators for LS, frailty and sarcopenia, with the Failure group showing lower performance across all three concepts compared with the Success group (Tables 1 and S1). When focusing on the frailty questionnaire items, significant differences were observed in five out of 15 items on the QMCOO, and in 11 out of 25 items on the KCL (Tables S2 and S3).

**Table 1:** Group comparison in the overall population according to whether the polyethylene terephthalate bottle cap can be opened.

	Total (*n* = 341)	Success group (*n* = 265)	Failure group (*n* = 76)	*P*‐value
Age (years)	80.0 ± 6.9	78.6 ± 6.6	84.8 ± 5.7	<0.001
Sex (female)	281 (82.4)	212 (80.0)	69 (90.8)	0.029
Height (cm)	151.0 ± 8.3	152.2 ± 8.1	146.9 ± 7.7	<0.001
Weight (kg)	54.0 ± 10.2	55.3 ± 9.9	49.5 ± 10.1	<0.001
Body mass index (kg/m^2^)	23.6 ± 3.5	23.8 ± 3.4	22.8 ± 3.7	0.030
Presence of LS				
LS stage 1, 2 and 3	300 (88.0)	226 (85.3)	74 (97.4)	0.004
LS stage 2 and 3	148 (43.4)	96 (36.2)	52 (68.4)	<0.001
LS Stage 3	45 (13.2)	22 (8.3)	23 (30.3)	<0.001
Stand‐up test				
One leg 40 cm (fail)	274 (80.4)	201 (75.8)	73 (96.1)	<0.001
Both legs 20 cm (fail)	54 (15.8)	34 (12.8)	20 (26.3)	0.005
Both legs 30 cm (fail)	16 (4.7)	9 (3.4)	7 (9.2)	0.035
Two‐step test value	1.13 ± 0.19	1.16 ± 0.18	1.01 ± 0.19	<0.001
<1.3	270 (79.2)	198 (74.7)	72 (94.7)	<0.001
<1.1	138 (40.5)	89 (33.6)	49 (64.5)	<0.001
<0.9	40 (11.7)	20 (7.5)	20 (26.3)	<0.001
GLFS‐5 (≥ 6 points)	110 (32.3)	69 (26.0)	41 (53.9)	<0.001
GLFS‐5 (total points)	3.0 [1.0–7.0]	2.0 [0.0–6.0]	6.0 [3.0–9.0]	<0.001
Presence of frailty				
J‐CHS criteria^†^	24 (12.8)	9 (6.5)	15 (30.6)	<0.001
J‐CHS criteria (total points)	1.0 [0.0–2.0]	1.0 [0.0–1.0]	2.0 [1.0–3.0]	<0.001
Presence of sarcopenia	51 (15.0)	25 (9.4)	26 (34.2)	<0.001
Grip strength (kg)	22.9 ± 6.5	24.3 ± 6.4	18.0 ± 4.4	<0.001
Usual walking speed (m/s)	1.21 ± 0.28	1.26 ± 0.26	1.01 ± 0.27	<0.001
SMI (kg/m^2^)	6.40 ± 0.79	6.51 ± 0.77	6.04 ± 0.74	<0.001

*Note*: Data are presented as mean ± standard deviation, number (%) or median [25th percentile–75th percentile]. The significance level is set at 5%. GLFS‐5, 5‐question Geriatric Locomotive Function Scale; J‐CHS, Japanese version of the Cardiovascular Health Study; LS, locomotive syndrome; SMI, skeletal muscle mass index.

^†^ *n* = 188.

**Figure 2:**
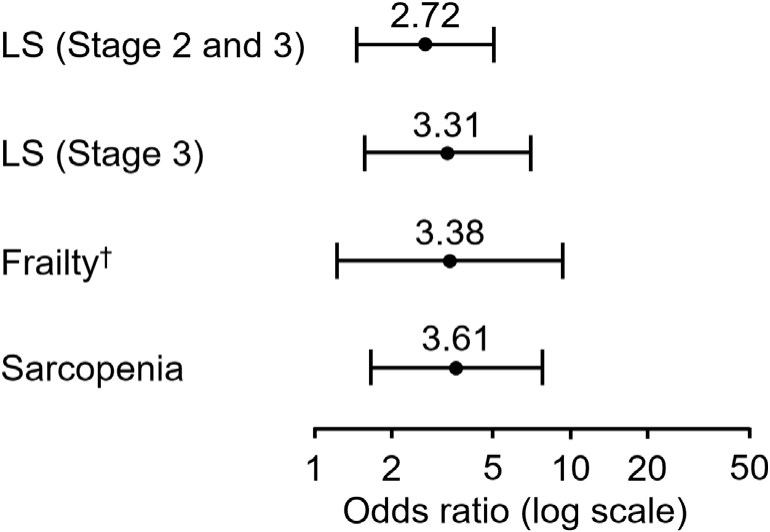
Odds ratios for the inability to open a polyethylene terephthalate bottle cap in relation to locomotive syndrome, frailty and sarcopenia in the overall population. This figure summarizes the results of four binomial logistic regression analyses. Frailty was assessed using the Japanese version of the Cardiovascular Health Study (J‐CHS) criteria. Dependent variables were absence of locomotive syndrome (LS)/frailty/sarcopenia = 0, and presence of LS/frailty/sarcopenia = 1. Independent variables were success group = 0, and failure group = 1. Adjusted variables were age, sex and body mass index. ^†^ *n* = 188. The significance level is set at 5%. J‐CHS, Japanese version of the Cardiovascular Health Study; LS, locomotive syndrome.

The results of the binary logistic regression analysis showed that the inability to open a PET bottle cap was associated with LS stages 2 and 3 (OR 2.72, 95% CI 1.46–5.07) and LS Stage 3 (OR 3.31, 95% CI 1.57–6.97; Fig. 2). However, the regression model was not significant when LS stages 1, 2 or 3 were included. Similarly, the presence of frailty was calculated for each assessment, showing significant associations with J‐CHS criteria (OR 3.38, 95% CI 1.23–9.24), the QMCOO (OR 2.37, 95% CI 1.31–4.29) and the KCL (OR 2.40, 95% CI 1.07–5.37; Fig. 2). Sarcopenia showed similar results (OR 3.61, 95% CI 1.67–7.79; Fig. 2).

Tables 2 and S4 list the sensitivity, specificity, PPV, negative predictive value and AUC for case finding using the PET bottle method to diagnose LS, frailty and sarcopenia. The ability involved in opening a PET bottle cap demonstrates a certain level of screening ability for LS, frailty and sarcopenia. Among these three concepts, the PET bottle method showed increased sensitivity and decreased specificity with a higher severity. Notably, the PPV was high: 97.4% for LS, 93.9% for pre‐frailty and frailty using the J‐CHS criteria, 69.7% using the QMCOO, and 89.8% using the KCL. The sex‐specific analyses are presented in Tables S5, S6 and S7, as well as in Figure S1. Figure S2 shows the ROC curve of each assessment value/score to distinguish whether or not a PET bottle cap can be opened.

**Table 2:** Sensitivity, specificity, positive and negative predictive values, and area under the curve of the screening based on whether a polyethylene terephthalate bottle cap can be opened for assessing locomotive syndrome, frailty and sarcopenia in the overall population.

	Sensitivity	Specificity	PPV	NPV	AUC (95% CI)	*P*‐value
Locomotive syndrome						
Stage 1, 2 and 3	24.7%	95.1%	97.4%	14.7%	0.599 (0.517–0.681)	0.040
Stage 2 and 3	35.1%	87.6%	68.4%	63.8%	0.613 (0.552–0.675)	<0.001
Stage 3	51.1%	82.1%	30.3%	91.7%	0.666 (0.574–0.759)	<0.001
Frailty (J‐CHS criteria)^†^						
Pre‐frailty and frailty	36.2%	95.1%	93.9%	41.7%	0.657 (0.579–0.734)	0.001
Frailty	62.5%	79.3%	30.6%	93.5%	0.709 (0.589–0.828)	0.001
Sarcopenia	51.0%	82.8%	34.2%	90.6%	0.669 (0.581–0.756)	<0.001
Severe sarcopenia	73.9%	81.4%	22.4%	97.7%	0.777 (0.670–0.884)	<0.001

*Note*: The significance level is set at 5%. AUC, area under the curve; CI, confidence interval; J‐CHS, Japanese version of the Cardiovascular Health Study; NPV, negative predictive value; PPV, positive predictive value.

^†^ *n* = 188.

## Discussion

In a previous study, we reported an association between the ability to open PET bottle caps and sarcopenia/frailty.^13^, ^14^, ^15^ Based on these findings, this is the first study to show that the ability to open PET bottle caps can be used to simultaneously and comprehensively assess LS, frailty and sarcopenia in community‐dwelling older adults. In addition, this study is the first to show an association between the ability required to open PET bottle caps and LS, as well as frailty assessed using the QMCOO. The results showed that the inability to open a PET bottle cap was associated with a 2.72‐fold higher risk of LS level ≥2, a 2.37‐ to 3.38‐fold higher risk of frailty and a 3.61‐fold higher risk of sarcopenia. In addition, the ability to open the PET bottle caps demonstrated a certain level of screening for LS (stages 2 and 3), frailty and sarcopenia. Notably, the PPV was high; among community‐dwelling older adults, 97.4% of those unable to open a PET bottle cap had LS. Furthermore, >70% of those unable to open a PET bottle cap were classified as having pre‐frailty or frailty according to the three main frailty assessments used in Japan. The high PPV indicates the potential for clinical application in efficiently detecting individuals at high risk of LS and frailty through medical interviews or other routine assessments.

The PET bottle method used in this study has several advantages. First, it provides a comparable or superior screening ability to existing screening methods for sarcopenia and frailty. The screening performance of our PET bottle method for sarcopenia showed a sensitivity of 51.0–73.9%, PPV of 22.4–34.2% and AUC of 0.669–0.777. In contrast, studies describing the screening performance for sarcopenia showed that calf circumference had a sensitivity of 62.9–79.2%, PPV of 6.1–20.8% and AUC of 0.613–0.684; the SARC‐F had a sensitivity of 10.4–23.9%, PPV of 10.6–25.6% and AUC of 0.516–0.585; and the SARC‐CalF had a sensitivity of 42.6–63.4%, PPV of 8.2–23.6% and AUC of 0.597–0.694.^29^ In another study, the AUC for detecting pre‐frailty by classifying 25 predictor variables into nine factors was 0.64.^30^ Second, the PET bottle method is easier to implement than existing screening methods. Because it does not require specialized equipment, difficulties in opening a PET bottle cap can be recognized by the older adults themselves, as well as by family members or friends in everyday life. Additionally, this method holds promise for use in medical interviews by medical doctors or healthcare professionals in settings without specialized equipment and for population‐based risk assessment at community events.^31^ Third, the ability required to open a cap reflects not only physical functions, such as muscle strength, but also the multifaceted nature of frailty, including mental, psychological and social aspects.^13^, ^14^, ^15^ In the present study, comparisons between the Success and Failure groups reflected all the assessment indicators of LS, frailty and sarcopenia. Notably, similar results were observed in both the phenotype (J‐CHS) and comprehensive models (QMCOO and KCL), which support an association between the ability required to open a PET bottle cap and frailty.

Although the PET bottle method shows promise, caution is warranted when using it as a standalone screening tool for LS. The PPV for LS was very high (97.4%), indicating that individuals who fail the PET bottle test are highly likely to have LS. However, the negative predictive value was low (14.7%), suggesting that passing the test does not necessarily rule out the presence of LS. Multivariate analysis showed a significant association between the inability to open a bottle cap and having LS stages 2 and 3. Based on these findings and other analytical results, the PET bottle cap‐opening ability is recommended as a screening tool for identifying individuals with LS stages 2 and 3.

On a different note, in this study's ROC curve of the subanalysis, we calculated the cutoff values for each assessment value/score to determine whether a PET bottle cap can be opened. A normal walking speed of 1.05 m/s, along with a grip strength of 17.7 kg from our previous study, approximated the cutoff values established by the Asian Working Group on Sarcopenia 2019.^6^, ^13^ The inability to open a PET bottle cap indicated low muscle strength and physical function. The cutoff value for the two‐step test was 1.1, which corresponds to the cutoff for LS stage 2. Integrating the findings from previous and current studies, when translating LS stages into more understandable terms for daily life, individuals with LS stage 2 were unable to open a PET bottle cap, and those with LS Stage 3 required walking aids for mobility.^32^ Thus, the ability required to open a PET bottle cap can help visualize the phases of LS or sarcopenia to which an individual might correspond.

The present study had some limitations. First, because this study used a cross‐sectional design, causal relationships cannot be inferred. To determine whether the ability to open PET bottle caps can predict the progression of LS, frailty or sarcopenia, as well as the future onset of care dependency, longitudinal studies will be necessary. Second, this study used a single type of 525‐mL PET bottle; however, cap specifications and designs can vary by brand and whether the product is domestic or international. Our previous study showed that the ability or inability to open the PET bottle cap was consistent across two different products. Third, there was a disparity in the number of male and female participants. In assessing sarcopenia and frailty, grip strength cutoff values differ between men and women, indicating potential sex‐related differences in screening performance. Although the robustness of the findings in older women was supported by the results presented in the Supporting Information, the sample size for men was insufficient to carry out all planned analyses. To ensure the robustness of the findings in older men, it is crucial to increase the sample size of male participants in future studies. Fourth, this study used the GLFS‐5, a simplified version of the 25‐question Geriatric Locomotive Function Scale (GLFS‐25). However, the GLFS‐5 has been validated against the GLFS‐25.^18^ Our previous research on the association between the ability to open PET bottles and sarcopenia/grip strength was cited in a subsequent study by another group that focused on cap‐grasping patterns, highlighting the growing interest in PET bottle‐opening research in Japan.^33^ The PET bottle methods are “simple” and “one action,” making them an easy and practical screening tool for LS, frailty and sarcopenia, with the potential for widespread social implementation.

## Conclusions

The present study is the first to develop a method for comprehensive and simultaneous assessment of LS, frailty and sarcopenia. The results showed that the ability to open a PET bottle cap is useful for screening LS (stages 2 and 3), frailty and sarcopenia. A notable finding was that the PPV was high, with >70% of older adults who were unable to open the PET bottle cap classified as having pre‐frailty or frailty.

## Disclosure statement

The authors declare no conflict of interest.

## Ethics statement

The study received approval from the Ethics Review Committee of the International University of Health and Welfare (Approval number: 18‐Io‐158‐2), and was conducted in accordance with the Declaration of Helsinki.

## Funding

This study was funded by JSPS Grants‐in‐Aid for Scientific Research (22 K17539 and 23 K06873) and a JGS Grant for Geriatric Nutrition Research supported by Otsuka Pharmaceutical Factory.

## Patient consent statement

After explaining the study via written and oral means to all participants, informed consent was obtained in writing.

## Supporting information


**Table S1.** Group comparison in the overall population according to whether the polyethylene terephthalate bottle cap can be opened.
**Table S2**. Group comparison in the overall population for the Questionnaire for Medical Checkup of Old‐Old by whether a polyethylene terephthalate bottle cap can be opened.
**Table S3**. Group comparison in the overall population for the Kihon Checklist based on whether a polyethylene terephthalate bottle cap can be opened.
**Table S4**. Sensitivity, specificity, positive and negative predictive values, and area under the curve of the screening based on whether a polyethylene terephthalate bottle cap can be opened for assessing frailty in the overall population.
**Table S5**. Group comparison in males according to whether the polyethylene terephthalate bottle cap can be opened.
**Table S6**. Group comparison in females according to whether the polyethylene terephthalate bottle cap can be opened.
**Table S7**. Sensitivity, specificity, positive and negative predictive values, and area under the curve of the screening based on whether a polyethylene terephthalate bottle cap can be opened for assessing locomotive syndrome, frailty, and sarcopenia in females.
**Figure S1**. Odds ratios for the inability to open a polyethylene terephthalate bottle cap in relation to locomotive syndrome, frailty, and sarcopenia in females.
**Figure S2**. Receiver operating characteristic curve of each assessment value/score in the overall population to distinguish whether or not a polyethylene terephthalate bottle cap can be opened.

## Data Availability

Research data were not shared because of privacy protection provisions of the City Hall.
