# Supplementary material for: Whether a polyethylene terephthalate bottle cap can be opened could serve as a common indicator for locomotive syndrome, frailty and sarcopenia
Source: Geriatr Gerontol Int. 2025 May 21;25(7):905–10. doi: 10.1111/ggi.70076 (PMC12238805; doi:10.1111/ggi.70076)
Supplement: Supplementary file 1 — Table S1. Group comparison in the overall population according to whether the polyethylene terephthalate bottle cap can be opened. Table S2. Group comparison in the overall population for the Questionnaire for Medical Checkup of Old‐Old by whether a polyethylene terephthalate bottle cap can be opened. Table S3. Group comparison in the overall population for the Kihon Checklist based on whether a polyethylene terephthalate bottle cap can be opened. Table S4. Sensitivity, specificity, positive and negative predictive values, and area under the curve of the screening based on whether a polyethylene terephthalate bottle cap can be opened for assessing frailty in the overall population. Table S5. Group comparison in males according to whether the polyethylene terephthalate bottle cap can be opened. Table S6. Group comparison in females according to whether the polyethylene terephthalate bottle cap can be opened. Table S7. Sensitivity, specificity, positive and negative predictive values, and area under the curve of the screening based on whether a polyethylene terephthalate bottle cap can be opened for assessing locomotive syndrome, frailty, and sarcopenia in females. Figure S1. Odds ratios for the inability to open a polyethylene terephthalate bottle cap in relation to locomotive syndrome, frailty, and sarcopenia in females. Figure S2. Receiver operating characteristic curve of each assessment value/score in the overall population to distinguish whether or not a polyethylene terephthalate bottle cap can be opened. [file GGI-25-905-s001.docx]

**Table S1.** Group comparison in the overall population according to whether the polyethylene terephthalate bottle cap can be opened

|  | Total  (*n*=341) | Success group (*n*=265) | Failure group (*n*=76) | *P* value |
| --- | --- | --- | --- | --- |
| LS stage |  |  |  |  |
| Non-LS | 41 (12.0) | 39 (14.7) | 2 (2.6) | **<0.001** |
| LS stage 1 | 152 (44.6) | 130 (49.1) | 22 (28.9) |  |
| LS stage 2 | 103 (30.2) | 74 (27.9) | 29 (38.2) |  |
| LS stage 3 | 45 (13.2) | 22 (8.3) | 23 (30.3) |  |
| Presence of frailty |  |  |  |  |
| QMCOO | 87 (25.5) | 54 (20.4) | 33 (43.4) | **<0.001** |
| QMCOO (total points) | 2.0 [1.0–4.0] | 2.0 [1.0–3.0] | 3.0 [2.0–5.0] | **<0.001** |
| KCL^†^ | 52 (27.5) | 27 (19.3) | 25 (51.0) | **<0.001** |
| KCL (total points) | 5.0 [2.0–8.0] | 4.0 [2.0–7.0] | 8.0 [5.0–10.0] | **<0.001** |

Data are presented as mean ± standard deviation, number (%), or median [25th percentile–75th percentile].

†: *n*=189.

The significance level is set at 5%.

KCL, Kihon Checklist; LS, locomotive syndrome; QMCOO, Questionnaire for Medical Checkup of Old-Old.

**Table S2.** Group comparison in the overall population for the Questionnaire for Medical Checkup of Old-Old by whether a polyethylene terephthalate bottle cap can be opened

| No. | Question | 1 point | Success group (*n*=265) | Failure group (*n*=76) | *P* value |
| --- | --- | --- | --- | --- | --- |
| 1 | How is your health condition? | Poor/Very poor | 14 (5.3) | 12 (15.8) | **0.002** |
| 2 | Are you satisfied with your daily life? | Moderately dissatisfied/ Dissatisfied | 9 (3.4) | 7 (9.2) | **0.035** |
| 3 | Do you eat three times a day? | No | 6 (2.3) | 3 (3.9) | 0.423 |
| 4 | Do you have any difficulties eating tough foods compared to 6 months ago? | Yes | 88 (33.2) | 31 (40.8) | 0.222 |
| 5 | Have you choked on your tea or soup recently? | Yes | 60 (22.6) | 20 (26.3) | 0.505 |
| 6 | Have you lost 2 kg or more in the past 6 months? | Yes | 28 (10.6) | 11 (14.5) | 0.345 |
| 7 | Do you think you walk slower than before? | Yes | 132 (49.8) | 56 (73.7) | **<0.001** |
| 8 | Have you experienced a fall in the past year? | Yes | 51 (19.2) | 21 (27.6) | 0.114 |
| 9 | Do you go for a walk for your health at least once a week? | No | 77 (29.1) | 31 (40.8) | 0.053 |
| 10 | Do your family or friends point out your memory loss? (e.g., “You ask the same question over and over again.”) | Yes | 41 (15.5) | 17 (22.4) | 0.158 |
| 11 | Do you find yourself not knowing today's date? | Yes | 66 (24.9) | 33 (43.4) | **0.002** |
| 12 | Do you smoke? | Yes | 13 (4.9) | 2 (2.6) | 0.536 |
| 13 | Do you go out at least once a week? | No | 6 (2.3) | 8 (10.5) | **0.001** |
| 14 | Do you keep regular communication with your family and friends? | No | 4 (1.5) | 3 (3.9) | 0.188 |
| 15 | When you are not feeling well, do you have anyone you can talk with? | No | 6 (2.3) | 1 (1.3) | 1.000 |

Data are presented as number (%). The significance level is set at 5%.

**Table S3.** Group comparison in the overall population for the Kihon Checklist based on whether a polyethylene terephthalate bottle cap can be opened

| No. | Question | 1 point | Success group (n=140) | Failure group (*n*=49) | *P* value |
| --- | --- | --- | --- | --- | --- |
| 1 | Do you go out by bus or train by yourself? | No | 19 (13.6) | 22 (44.9) | **<0.001** |
| 2 | Do you go shopping to buy daily necessities by yourself? | No | 4 (2.9) | 11 (22.4) | **<0.001** |
| 3 | Do you manage your own deposits and savings at the bank? | No | 10 (7.1) | 12 (24.5) | **0.001** |
| 4 | Do you sometimes visit your friends? | No | 12 (8.6) | 10 (20.4) | **0.026** |
| 5 | Do your family or friends turn to you for advice? | No | 10 (7.1) | 12 (24.5) | **0.001** |
| 6 | Do you normally climb stairs without using handrail or wall for support? | No | 45 (32.1) | 36 (73.5) | **<0.001** |
| 7 | Do you normally stand up from a chair without any aids? | No | 21 (15.0) | 20 (40.8) | **<0.001** |
| 8 | Do you normally walk continuously for 15 minutes? | No | 7 (5.0) | 10 (20.4) | **0.001** |
| 9 | Have you experienced a fall in the past year? | Yes | 34 (24.3) | 16 (32.7) | 0.253 |
| 10 | Do you have a fear of falling while walking? | Yes | 74 (52.9) | 35 (71.4) | **0.024** |
| 11 | Have you lost 2 kg or more in the past 6 months? | Yes | 24 (17.1) | 13 (26.5) | 0.154 |
| 12 | Height: cm, Weight: kg, BMI: kg/m^2^  If BMI is less than 18.5, this item is scored. | Yes | 7 (5.0) | 5 (10.2) | 0.199 |
| 13 | Do you have any difficulties eating tough foods compared to 6 months ago? | Yes | 44 (31.4) | 17 (34.7) | 0.674 |
| 14 | Have you choked on your tea or soup recently? | Yes | 31 (22.1) | 11 (22.4) | 0.965 |
| 15 | Do you often experience having a dry mouth? | Yes | 38 (27.1) | 13 (26.5) | 0.934 |
| 16 | Do you go out at least once a week? | No | 4 (2.9) | 5 (10.2) | 0.052 |
| 17 | Do you go out less frequently compared to last year? | Yes | 35 (25.0) | 24 (49.0) | **0.002** |
| 18 | Do your family or your friends point out your memory loss? e.g., "You ask the same question over and over again." | Yes | 24 (17.1) | 9 (18.4) | 0.846 |
| 19 | Do you make a call by looking up phone numbers? | No | 8 (5.7) | 1 (2.0) | 0.450 |
| 20 | Do you find yourself not knowing today’s date? | Yes | 42 (30.0) | 25 (51.0) | **0.008** |
| 21 | In the last 2 weeks have you felt a lack of fulfillment in your daily life? | Yes | 20 (14.3) | 9 (18.4) | 0.495 |
| 22 | In the last 2 weeks have you felt a lack of joy when doing the things you used to enjoy? | Yes | 12 (8.6) | 5 (10.2) | 0.731 |
| 23 | In the last 2 weeks have you felt difficulty in doing what you could do easily before? | Yes | 53 (37.9) | 24 (49.0) | 0.173 |
| 24 | In the last 2 weeks have you felt helpless? | Yes | 25 (17.9) | 13 (26.5) | 0.192 |
| 25 | In the last 2 weeks have you felt tired without a reason? | Yes | 38 (27.1) | 20 (40.8) | 0.074 |

Data are presented as number (%). The significance level is set at 5%. BMI, body mass index.

**Table S4.** Sensitivity, specificity, positive and negative predictive values, and area under the curve of the screening based on whether a polyethylene terephthalate bottle cap can be opened for assessing frailty in the overall population

|  | Sensitivity | Specificity | PPV | NPV | AUC (95% CI) | *P* value |
| --- | --- | --- | --- | --- | --- | --- |
| Frailty |  |  |  |  |  |  |
| QMCOO |  |  |  |  |  |  |
| Pre-frailty and frailty | 33.8% | 87.5% | 69.7% | 60.8% | 0.606 (0.546–0.667) | **0.001** |
| Frailty | 37.9% | 83.1% | 43.4% | 79.6% | 0.605 (0.533–0.677) | **0.003** |
| KCL^†^ |  |  |  |  |  |  |
| Pre-frailty and frailty | 36.4% | 92.6% | 89.8% | 45.0% | 0.645 (0.567–0.723) | **0.001** |
| Frailty | 48.1% | 82.5% | 51.0% | 80.7% | 0.653 (0.560–0.745) | **0.001** |

†: *n*=189.

The significance level is set at 5%.

AUC, Area under the curve; CI, confidence interval; KCL, Kihon Checklist; NPV, negative predictive value; PPV, positive predictive value; QMCOO, Questionnaire for Medical Checkup of Old-Old.

**Table S5.** Group comparison in males according to whether the polyethylene terephthalate bottle cap can be opened

|  | Total  (*n*=60) | Success group (*n*=53) | Failure group (*n*=7) | *P* value |
| --- | --- | --- | --- | --- |
| Age | 77.9 ± 6.9 | 76.8 ± 6.2 | 86.0 ± 7.0 | **0.001** |
| Height (cm) | 162.4 ± 7.0 | 163.0 ± 7.0 | 158.2 ± 5.5 | 0.086 |
| Weight (kg) | 65.0 ± 10.4 | 64.9 ± 10.5 | 66.0 ± 10.5 | 0.793 |
| Body mass index (kg/m^2^) | 24.6 ± 3.5 | 24.4 ± 3.3 | 26.5 ± 5.2 | 0.136 |
| Presence of LS |  |  |  |  |
| LS stage 1, 2, and 3 | 47 (78.3) | 40 (75.5) | 7 (100.0) | 0.329 |
| LS stage 2 and 3 | 25 (41.7) | 21 (39.6) | 4 (57.1) | 0.436 |
| LS stage 3 | 9 (15.0) | 6 (11.3) | 3 (42.9) | 0.062 |
| Stand-up test |  |  |  |  |
| One leg 40 cm (fail) | 42 (70.0) | 35 (66.0) | 7 (100.0) | 0.091 |
| Both legs 20 cm (fail) | 9 (15.0) | 7 (13.2) | 2 (28.6) | 0.281 |
| Both legs 30 cm (fail) | 3 (5.0) | 3 (5.7) | 0 (0.0) | 1.000 |
| Two-step test value | 1.15 ± 0.21 | 1.17 ± 0.20 | 0.99 ± 0.18 | **0.030** |
| <1.3 | 40 (66.7) | 33 (62.3) | 7 (100.0) | 0.084 |
| <1.1 | 22 (36.7) | 18 (34.0) | 4 (57.1) | 0.405 |
| <0.9 | 8 (13.3) | 5 (9.4) | 3 (42.9) | **0.043** |
| GLFS-5 (≥ 6 points) | 16 (26.7) | 11 (20.8) | 5 (71.4) | **0.012** |
| GLFS-5 (total points) | 2.0 [0.0–6.8] | 1.0 [0.0–4.5] | 9.0 [3.0–10.0] | **0.008** |
| Presence of frailty |  |  |  |  |
| J-CHS criteria^†^ | 4 (11.8) | 3 (10.0) | 1 (25.0) | 0.409 |
| J-CHS criteria (total points) | 1.0 [0.0–2.0] | 1.0 [0.0–1.3] | 2.0 [2.0–3.5] | **0.013** |
| QMCOO | 14 (23.3) | 10 (18.9) | 4 (57.1) | **0.045** |
| QMCOO (total points) | 2.0 [1.0–3.0] | 2.0 [1.0–3.0] | 4.0 [3.0–5.0] | **0.001** |
| KCL^†^ | 6 (17.6) | 3 (10.0) | 3 (75.0) | **0.012** |
| KCL (total points) | 3.5 [1.0–7.0] | 2.5 [1.0–7.0] | 8.5 [5.0–9.8] | **0.013** |
| Presence of sarcopenia | 17 (28.3) | 12 (22.6) | 5 (71.4) | **0.016** |
| Grip strength (kg) | 32.2 ± 7.4 | 33.3 ± 7.1 | 24.0 ± 3.8 | **0.001** |
| Usual walking speed (m/sec) | 1.22 ± 0.26 | 1.25 ± 0.25 | 1.04 ± 0.28 | **0.045** |
| SMI (kg/m^2^) | 7.30 ± 0.96 | 7.37 ± 0.98 | 6.78 ± 0.55 | 0.128 |

Data are presented as mean ± standard deviation, number (%), or median [25th percentile–75th percentile].

†: *n*=34.

The significance level is set at 5%.

GLFS-5, 5-question Geriatric Locomotive Function Scale; J-CHS, Japanese version of the Cardiovascular Health Study; KCL, Kihon Checklist; LS, locomotive syndrome; QMCOO, Questionnaire for Medical Checkup of Old-Old; SMI, skeletal muscle mass index.

**Table S6.** Group comparison in females according to whether the polyethylene terephthalate bottle cap can be opened

|  | Total  (*n*=281) | Success group (*n*=212) | Failure group (*n*=69) | *P* value |
| --- | --- | --- | --- | --- |
| Age | 80.5 ± 6.8 | 79.1 ± 6.6 | 84.7 ± 5.6 | **<0.001** |
| Height (cm) | 148.5 ± 6.3 | 149.4 ± 5.8 | 145.8 ± 6.9 | **<0.001** |
| Weight (kg) | 51.6 ± 8.5 | 52.9 ± 8.2 | 47.8 ± 8.5 | **<0.001** |
| Body mass index (kg/m^2^) | 23.4 ± 3.5 | 23.7 ± 3.5 | 22.5 ± 3.4 | **0.010** |
| Presence of LS |  |  |  |  |
| LS stage 1, 2, and 3 | 253 (90.0) | 186 (87.7) | 67 (97.1) | **0.024** |
| LS stage 2 and 3 | 123 (43.8) | 75 (35.4) | 48 (69.6) | **<0.001** |
| LS stage 3 | 36 (12.8) | 16 (7.5) | 20 (29.0) | **<0.001** |
| Stand-up test |  |  |  |  |
| One leg 40 cm (fail) | 232 (82.6) | 166 (78.3) | 66 (95.7) | **0.001** |
| Both legs 20 cm (fail) | 45 (16.0) | 27 (12.7) | 18 (26.1) | **0.009** |
| Both legs 30 cm (fail) | 13 (4.6) | 6 (2.8) | 7 (10.1) | **0.012** |
| Two-step test value | 1.13 ± 0.19 | 1.16 ± 0.18 | 1.02 ± 0.20 | **<0.001** |
| <1.3 | 230 (81.9) | 165 (77.8) | 65 (94.2) | **0.002** |
| <1.1 | 116 (41.3) | 71 (33.5) | 45 (65.2) | **<0.001** |
| <0.9 | 32 (11.4) | 15 (7.1) | 17 (24.6) | **<0.001** |
| GLFS-5 (≥ 6 points) | 94 (33.5) | 58 (27.4) | 36 (52.2) | **<0.001** |
| GLFS-5 (total points) | 3.0 [1.0–7.0] | 2.5 [1.0–6.0] | 6.0 [3.0–9.0] | **<0.001** |
| Presence of frailty |  |  |  |  |
| J-CHS criteria^†^ | 20 (13.0) | 6 (5.5) | 14 (31.1) | **<0.001** |
| J-CHS criteria (total points) | 1.0 [0.0–2.0] | 1.0 [0.0–1.0] | 2.0 [1.0–3.0] | **<0.001** |
| QMCOO | 73 (26.0) | 44 (20.8) | 29 (42.0) | **<0.001** |
| QMCOO (total points) | 2.0 [1.0–4.0] | 2.0 [1.0–3.0] | 3.0 [2.0–4.5] | **<0.001** |
| KCL^‡^ | 46 (29.7) | 24 (21.8) | 22 (48.9) | **0.001** |
| KCL (total points) | 5.0 [3.0–9.0] | 4.0 [2.0–7.0] | 7.0 [5.0–10.0] | **<0.001** |
| Presence of sarcopenia | 34 (12.1) | 13 (6.1) | 21 (30.4) | **<0.001** |
| Grip strength (kg) | 20.9 ± 4.2 | 22.0 ± 3.6 | 17.4 ± 4.0 | **<0.001** |
| Usual walking speed (m/sec) | 1.20 ± 0.28 | 1.27 ± 0.26 | 1.01 ± 0.27 | **<0.001** |
| SMI (kg/m^2^) | 6.21 ± 0.59 | 6.29 ± 0.52 | 5.96 ± 0.72 | **0.001** |

Data are presented as mean ± standard deviation, number (%), or median [25th percentile–75th percentile].

†: *n*=154, ‡: *n*=155.

The significance level is set at 5%.

GLFS-5, 5-question Geriatric Locomotive Function Scale; J-CHS, Japanese version of the Cardiovascular Health Study; KCL, Kihon Checklist; LS, locomotive syndrome; QMCOO, Questionnaire for Medical Checkup of Old-Old; SMI, skeletal muscle mass index.

**Table S7.** Sensitivity, specificity, positive and negative predictive values, and area under the curve of the screening based on whether a polyethylene terephthalate bottle cap can be opened for assessing locomotive syndrome, frailty, and sarcopenia in females

|  | Sensitivity | Specificity | PPV | NPV | AUC (95% CI) | *P* value |
| --- | --- | --- | --- | --- | --- | --- |
| Locomotive syndrome |  |  |  |  |  |  |
| Stage 1, 2, and 3 | 26.5% | 92.9% | 97.1% | 12.3% | 0.597 (0.498–0.695) | 0.093 |
| Stage 2 and 3 | 39.0% | 86.7% | 69.6% | 64.6% | 0.629 (0.562–0.696) | **<0.001** |
| Stage 3 | 55.6% | 80.0% | 29.0% | 92.5% | 0.678 (0.576–0.779) | **0.001** |
| Frailty |  |  |  |  |  |  |
| J-CHS criteria^†^ |  |  |  |  |  |  |
| Pre-frailty and frailty | 39.3% | 93.6% | 93.3% | 40.4% | 0.664 (0.578–0.751) | **0.001** |
| Frailty | 70.0% | 76.9% | 31.1% | 94.5% | 0.734 (0.611–0.858) | **0.001** |
| QMCOO |  |  |  |  |  |  |
| Pre-frailty and frailty | 34.8% | 84.6% | 66.7% | 59.4% | 0.597 (0.530–0.664) | **0.005** |
| Frailty | 39.7% | 80.8% | 42.0% | 79.2% | 0.602 (0.524–0.681) | **0.009** |
| KCL^‡^ |  |  |  |  |  |  |
| Pre-frailty and frailty | 38.5% | 90.2% | 88.9% | 41.8% | 0.643 (0.556–0.731) | **0.004** |
| Frailty | 47.8% | 78.9% | 48.9% | 78.2% | 0.634 (0.534–0.733) | **0.009** |
| Sarcopenia | 61.8% | 80.6% | 30.4% | 93.9% | 0.712 (0.611–0.812) | **<0.001** |
| Severe sarcopenia | 87.5% | 79.2% | 20.3% | 99.1% | 0.834 (0.735–0.932) | **<0.001** |

†: n=154, ‡: n=155.

The significance level is set at 5%.

AUC, Area under the curve; CI, confidence interval; J-CHS, Japanese version of the Cardiovascular Health Study; KCL, Kihon Checklist; NPV, negative predictive value; PPV, positive predictive value; QMCOO, Questionnaire for Medical Checkup of Old-Old.


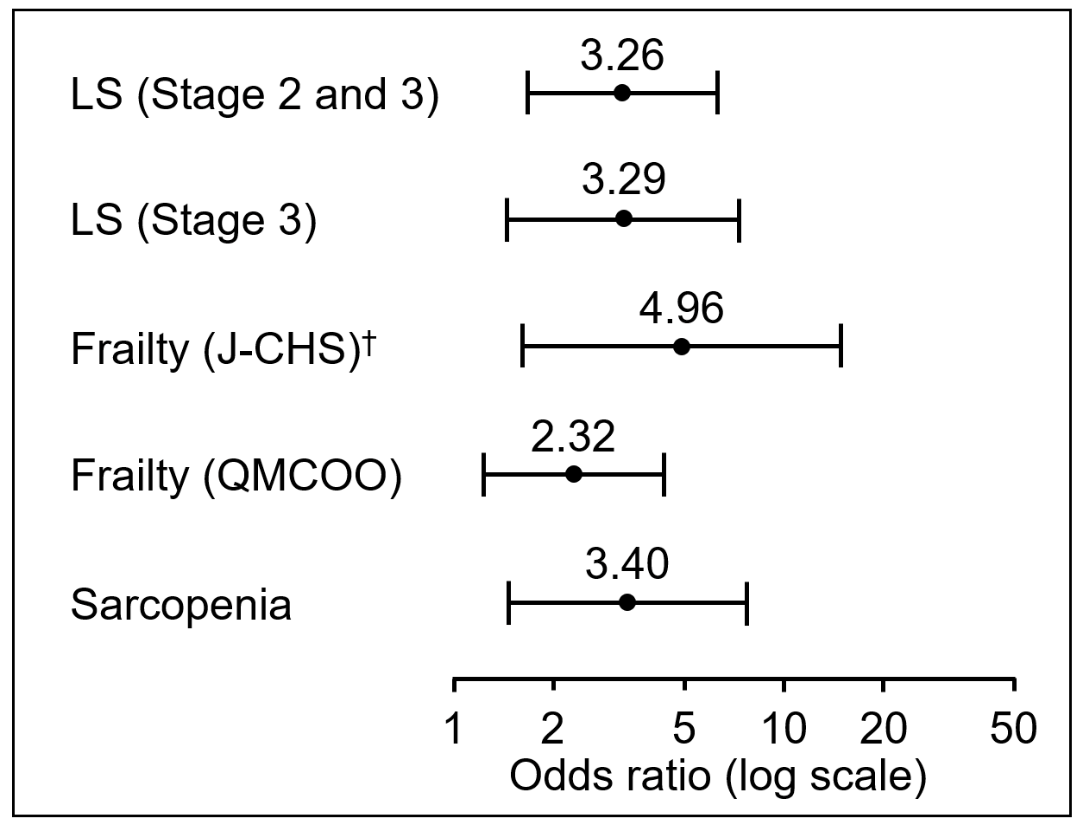


**Figure S1.** Odds ratios for the inability to open a polyethylene terephthalate bottle cap in relation to locomotive syndrome, frailty, and sarcopenia in females

This figure summarizes the results of five binomial logistic regression analyses. Frailty, as assessed using the Kihon Checklist, was not found to be associated with the inability to open a PET bottle cap.

Dependent variable: Absence of LS/frailty/sarcopenia=0, Presence of LS/frailty/sarcopenia=1.

Independent variables: Success group=0, Failure group=1.

Adjusted variables: Age and body mass index.

†: n=154.

The significance level is set at 5%.

J-CHS, Japanese version of the Cardiovascular Health Study; LS, locomotive syndrome; QMCOO, Questionnaire for Medical Checkup of Old-Old.


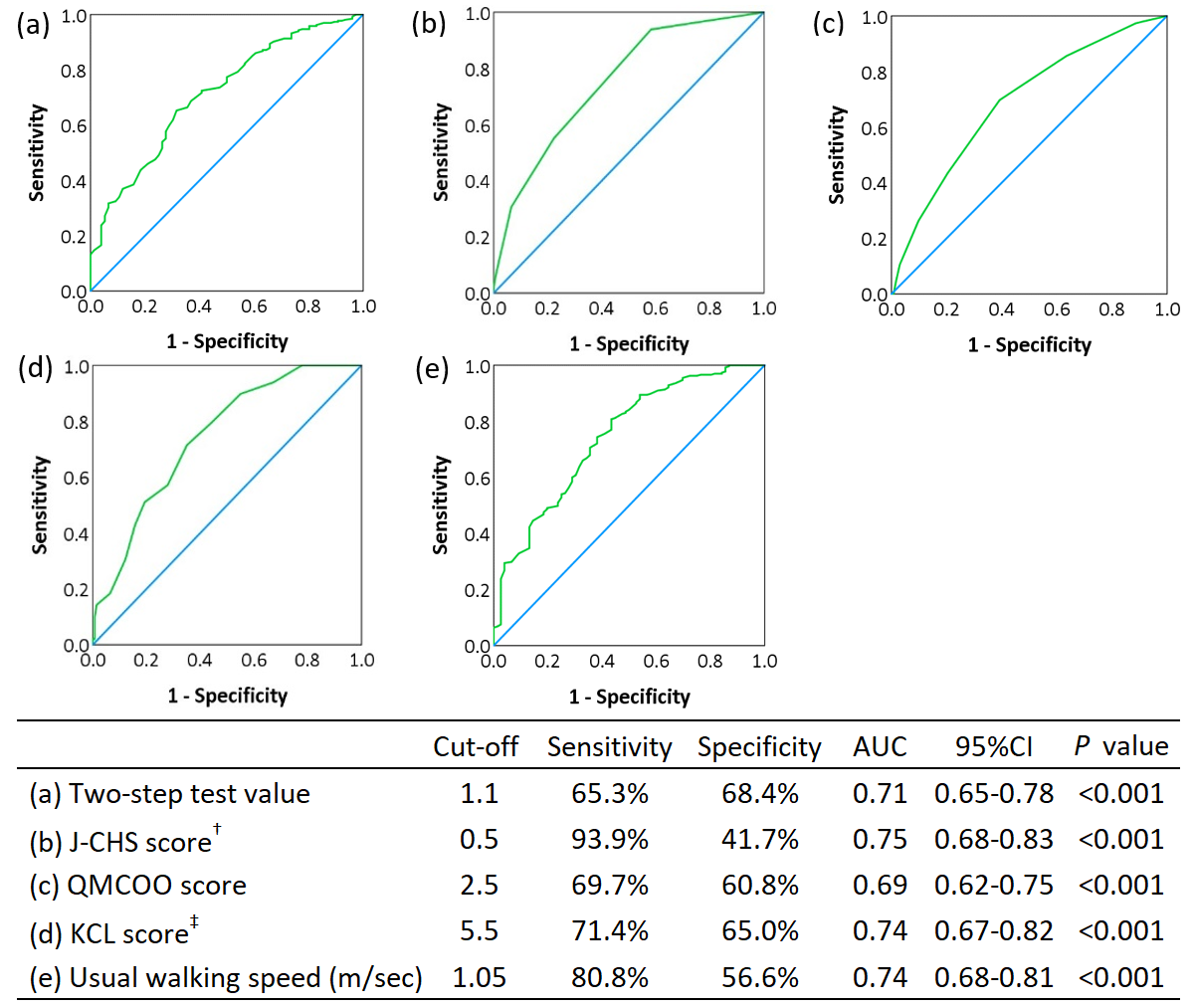


**Figure S2.** Receiver operating characteristic curve of each assessment value/score in the overall population to distinguish whether or not a polyethylene terephthalate bottle cap can be opened

†: *n*=188, ‡: *n*=189.

The significance level is set at 5%.

AUC, Area under the curve; CI, confidence interval; J-CHS, Japanese version of the Cardiovascular Health Study; KCL, Kihon Checklist; QMCOO, Questionnaire for Medical Checkup of Old-Old.
